# Incidence and determinants of adverse events in individuals with HIV commencing Dolutegravir-based antiretroviral therapy in mainland Tanzania

**DOI:** 10.1038/s41598-023-51144-7

**Published:** 2024-01-05

**Authors:** Adam Fimbo, Yonah H. Mwalwisi, Kissa Mwamwitwa, Damas Matiko, Elirehema Mfinanga, Johnson Lyimo, Amon Sabasaba, Seth Missago, Elias Bukundi, Goodluck Gotora, Dorice Respick, Alex Nkayamba, Emmanuel Masunga, Rajabu Hussein Mnkugwe, Peter P. Kunambi, Castory Munishi, Christine Chiedza Musanhu, Omary M. S. Minzi, Eulambius M. Mlugu

**Affiliations:** 1 Tanzania Medicines and Medical Devices Authority (TMDA), Dodoma, Tanzania; 2 World Health Organization, Dar Es Salaam, Tanzania; 3 Department of Epidemiology, School of Public Health, Muhimbili University of Health and Allied Sciences, Dar Es Salaam, Tanzania; 4 National Institute for Medical Research, Headquarters, Dar Es Salaam, Tanzania; 5 Department of Clinical Pharmacology, School of Biomedical Sciences, Campus College of Medicine, Muhimbili University of Health and Allied Sciences, Dar Es Salaam, Tanzania; 6 Department of Pharmaceutics and Pharmacy Practice, School of Pharmacy, Muhimbili University of Health and Allied Sciences, Dar Es Salaam, Tanzania; 7 Department of Clinical Pharmacy and Pharmacology, School of Pharmacy, Muhimbili University of Health and Allied Sciences, Dar Es Salaam, Tanzania

**Keywords:** HIV infections, Viral infection

## Abstract

Tanzania adopted a Dolutegravir (DTG)-based regimen as first-line treatment in 2019 following the World Health Organization recommendation. Data on the DTG safety profile from sub-Saharan Africa including Tanzania are limited. We investigated the incidence of DTG-related adverse events (AEs) and associated factors among people living with HIV (PLHIV) initiated on a DTG regimen. A prospective cohort study was conducted from 25 Care and Treatment Clinics in mainland Tanzania. PLHIV aged 12 years and above who were initiated on a DTG-based regimen were actively followed up for three months. The Cox regression model was used to determine the predictors of occurrence of AEs over time. A *p*-value of 0.05 was considered statistically significant. From January 2020 to June 2022, a cohort of 935 participants who were both newly diagnosed and ART-experienced who transitioned to a DTG-based regimen was enrolled. Out of 935 participants, 59 (6.3%) reported a total of 62 AEs. The most frequently experienced AE was skin itching and rashes (15/62; 24.2%). DTG-associated neuropsychiatric AEs were less common and included headache (6 [9.6%]) and sleep disturbances (3 [4.8%]). The overall incidence of occurrence of the first AEs was 96.7 per 1000 person-months [95% C.I: 74.4–125.7] with the highest incidence observed among the elderly (≥ 60 years). Individuals on WHO HIV Clinical Stage 2 had a 2.7 significantly higher risk of developing AEs (adjusted hazard ratio = 2.73, 95% CI = 1.46–5.12, *p* = 0.017). We report a low incidence of grade I (mild) and grade II (moderate) DTG-associated AEs suggesting that the regimen is generally safe in the population. Continued monitoring of DTG safety in the population is recommended.

## Introduction

Globally, an estimated 39 million people were living with HIV/AIDS in 2022^1^. Like in many sub-Saharan African countries, HIV is still a major public health problem in Tanzania, with a prevalence of 4.7%, corresponding to 1.7 million people living with HIV/AIDS (PLHIV) as of 2021^2^. Since the introduction of Antiretroviral therapy (ART) in Tanzania in the early 2000s, over 1.5 million PLHIV have been initiated on ART, and 83% have attained viral load suppression and adequate immunity^2^. Consequently, this has reduced HIV-related complications, improved health, prolonged survival, and reduced risk of HIV transmission^3^. However, ARTs have toxicities and adverse reactions, which may lead to attrition of patients from care^4–6^. The incidence of ARTs-related adverse drug reactions (ADR) has been reported to range from 11 to 35.9% in both developed and developing countries^7^.

In 2014, the Joint United Nations Programme launched the 90-90-90 AIDS program which was later updated to 95-95-95 in 2020, targeting to diagnose 95% of all HIV-positive individuals, provide ART for 95% of those diagnosed and achieve viral suppression for 95% of those treated by 2030^8^. As of 2021, Tanzania has made significant progress towards achieving the 95-95-95 targets as indicated by the increased ART coverage to 86%^2,9^. Nevertheless, the achievement of these targets is affected by several factors including drug resistance and ADRs related to some ART drugs.

DTG is a second-generation integrase-strand transfer inhibitor (INSTI) with higher efficacy, excellent tolerability, infrequent drug-drug interactions and mild adverse events^2,10^. Due to its safety profile and fewer chances of developing resistance, the World Health Organization (WHO) recommended a DTG-based regimen to be the first line of ART to stimulate progress towards achieving the 95-95-95 targets^11,12^. Tanzania adopted a DTG-based regimen as a first and second-line regimen in 2019.

Studies done in real-world settings reported DTG-associated neuropsychiatric adverse reactions and increased risk of neural tube defects among infants^13–18^. These AEs may negatively impact ART adherence leading to discontinuation, therapeutic failure, and viral resistance interrupting the achievement of 95-95-95 AIDS targets^19–21^. Data on the DTG safety profile from sub-Saharan African countries are limited^7,9,22^. A large study conducted in six Eastern-southern African countries including Tanzania, reported that a DTG based regimen was safe with mild AEs among children and adolescents^23^. Similarly, mild to moderate AEs were reported among children and adolescents attending CTC in the Mbeya region, mainland Tanzania^24^. While adults comprise the majority of PLHIV, data on DTG safety profiles among adults from Tanzania are not available to the best of our knowledge. The present study reports the incidence of AEs and risk factors among adolescents and adults PLHIV initiated on a DTG based regimen from 11 regions of mainland Tanzania.

## Results

### Baseline characteristics of study participants

**Figure 1:**
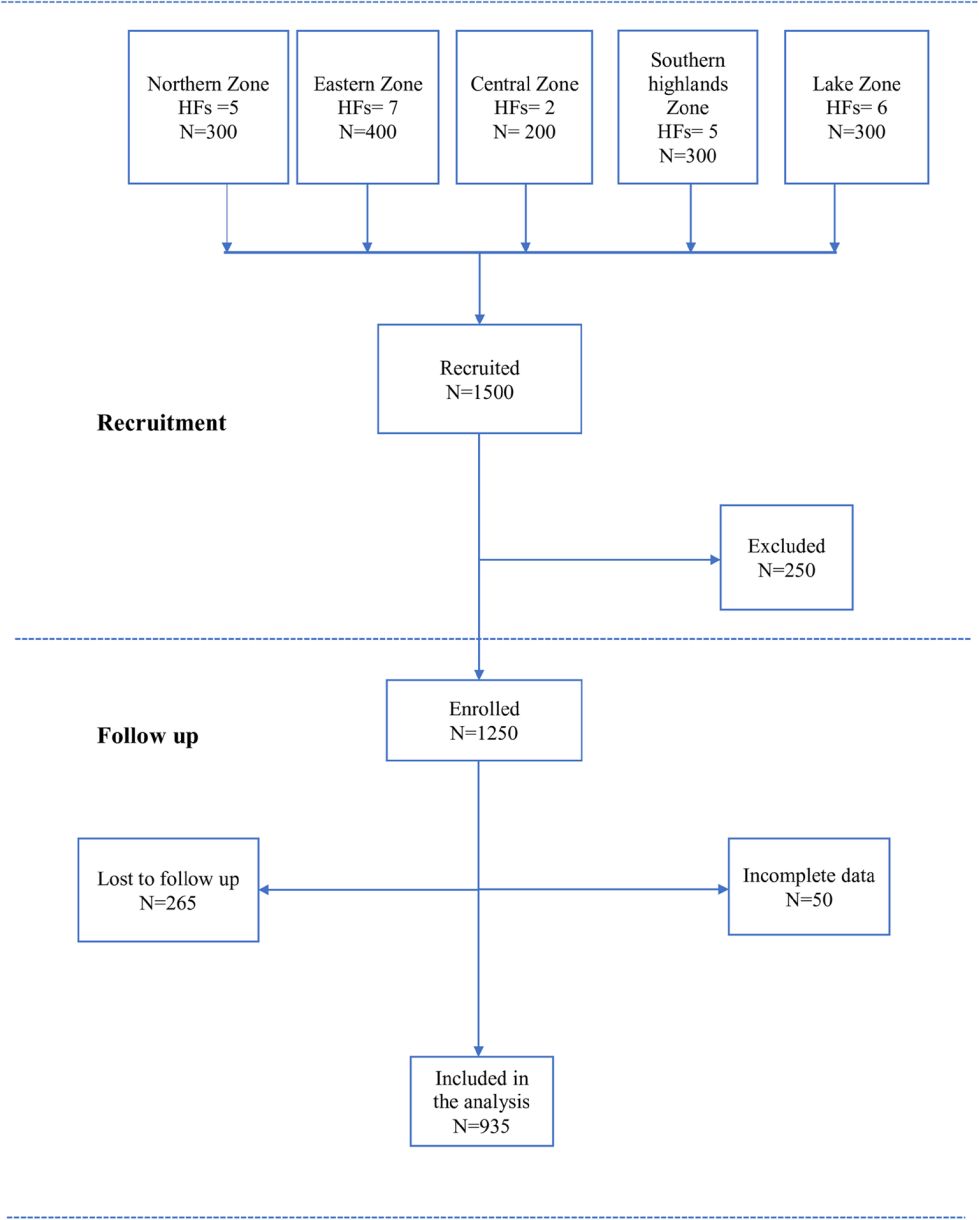
Study flow chart. HFs = Health facilities.

Figure 1 indicates the number of individuals recruited from each administrative zone and the corresponding total number of those followed up to 90 days. Among 1500 individuals recruited, 250 participants were excluded at enrollment due to age (< 12 years) or serious illness that needed hospitalization. From January 2020 to June 2022, a total of 935 participants were followed up for 90 days (Fig. 1).

The majority of participants were females (591) (63.2%). More than eighty per cent were aged between 25 and 59 years (763/935; 81.6%), with the median age (IQR) of the study population being 35 (28, 44) years. At baseline, the majority of participants had a body weight of more than 40 kg (882/935; 94.3%), and about two-thirds of them were categorized as WHO HIV clinical stage I (628/935; 67.2%). Participants with a CD4 cell count of ≥ 500 cells/mm^3^ accounted for the majority (436/935; 46.6%). Table 1 presents the baseline characteristics of the study population.

**Table 1:** Baseline characteristics of study participants.

Variable	Frequency (n)	Percent (%)
Age group (years)		
12–19	26	2.8
20–24	101	10.8
25–59	763	81.6
> 60	45	4.8
Median age in years (IQR)	35 (28, 44)	
Sex		
Male	344	36.8
Female	591	63.2
Marital status		
Single	249	26.6
Cohabiting	38	4.1
Married	457	48.9
Divorced	98	10.5
Widow/separated	93	9.9
Education level		
No formal education	90	9.6
Primary education	610	65.2
Secondary education	200	21.4
College and University	35	3.7
Body weight (kg)		
25–40	53	5.7
> 40	882	94.3
Median weight in Kg (IQR)	57.00 (50.00, 63.40)	
Absolute CD4 count (cells/mm^3^)		
< 50	3	0.3
50– < 200	196	21
200– < 500	718	32.1
≥ 500	436	46.6
Median CD4 count in cells/ mm^3^ (IQR)	401 (230, 585)	
WHO HIV Clinical stages		
Stage I	628	67.2
Stage II	173	18.5
Stage III	98	10.5
Stage IV	36	3.9

### Reported adverse events (AEs)

**Figure 2:**
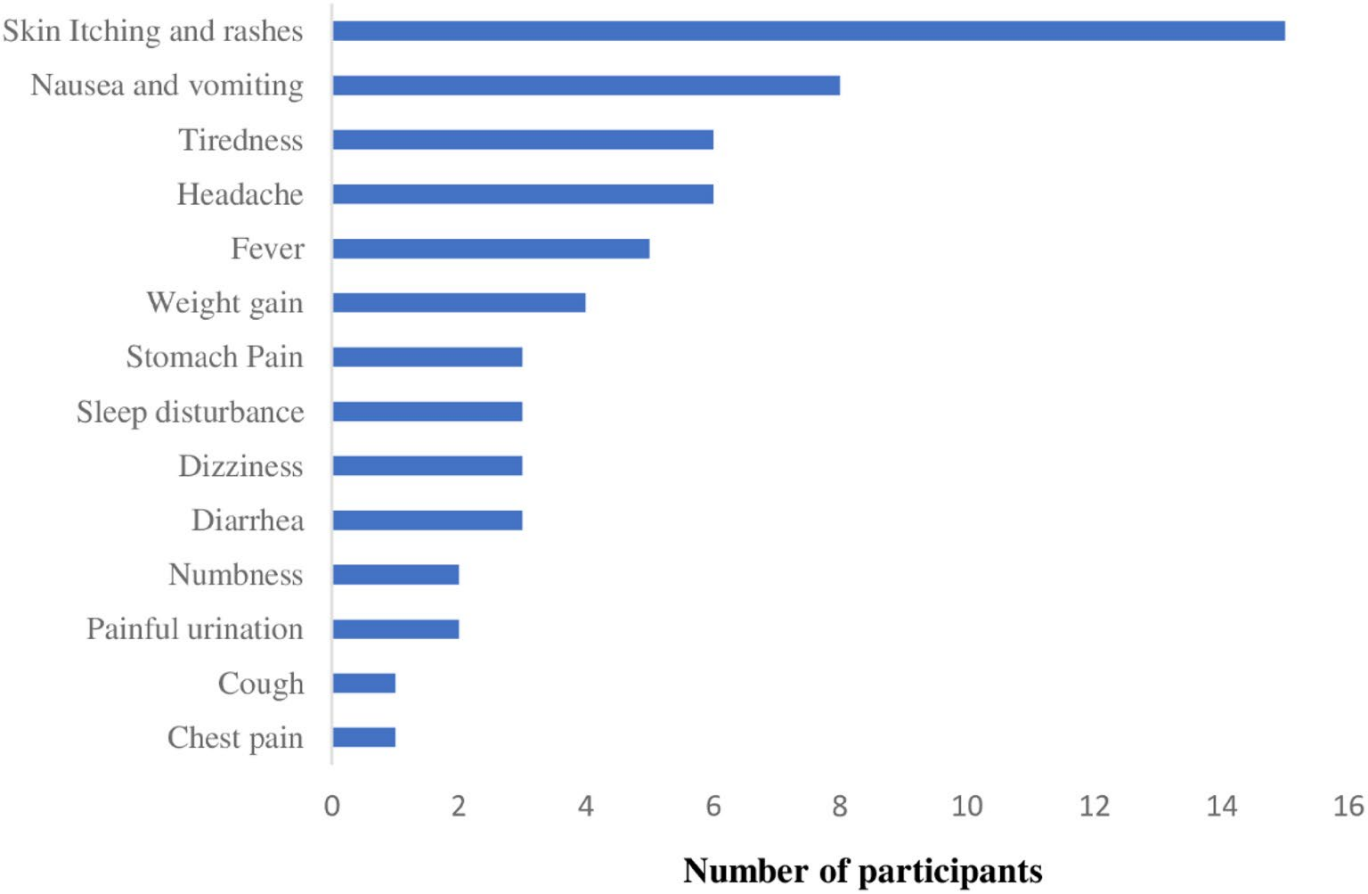
Adverse events reported within 90 days following initiation of DTG based-regimen.

Out of 935 participants, 59 (6.3%) reported experiencing at least one AE with a total of 62 AEs (Fig. 2). Three participants (0.3%) reported experiencing two AEs at the same encounter. The most frequently reported AEs were skin itching and rashes (15) (1.6%) followed by nausea and vomiting (8) (0.9%); tiredness (6) (0.6%); headache (6) (0.6%) and fever (5) (0.5%). The least reported AEs were numbness (2) (0.2%); painful urination (2) (0.2%); cough (1) (0.1%) and chest pain (1) (0.1%) (Fig. 2). All the reported AEs were mild (grade I) to moderate (grade II) according to the internationally regognised Common Terminology Criteria for Adverse Events (CTCAE).

**Figure 3:**
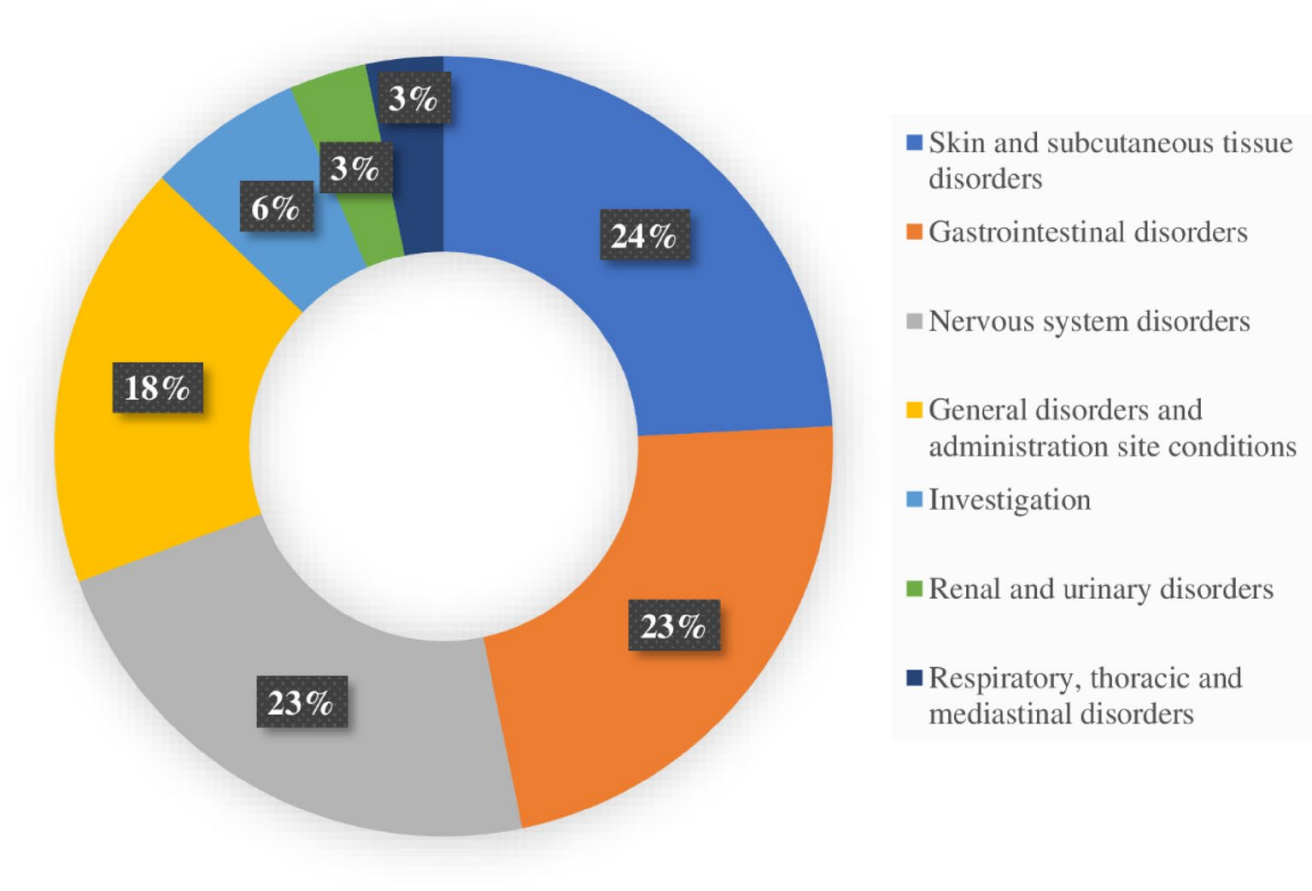
System Organ Classification (SOC) of the reported adverse events within 90 days following initiation of TLD.

The reported AEs fell within 7 out of 27 MedDRA System Organ Classes (SOC). Skin and Subcutaneous tissue disorders accounted for the majority (24%) followed by gastrointestinal disorders (23%), nervous system disorders (23%), general disorders and administrative site conditions (18%) (Fig. 3).

### Incidence rates of adverse events

**Figure 4:**
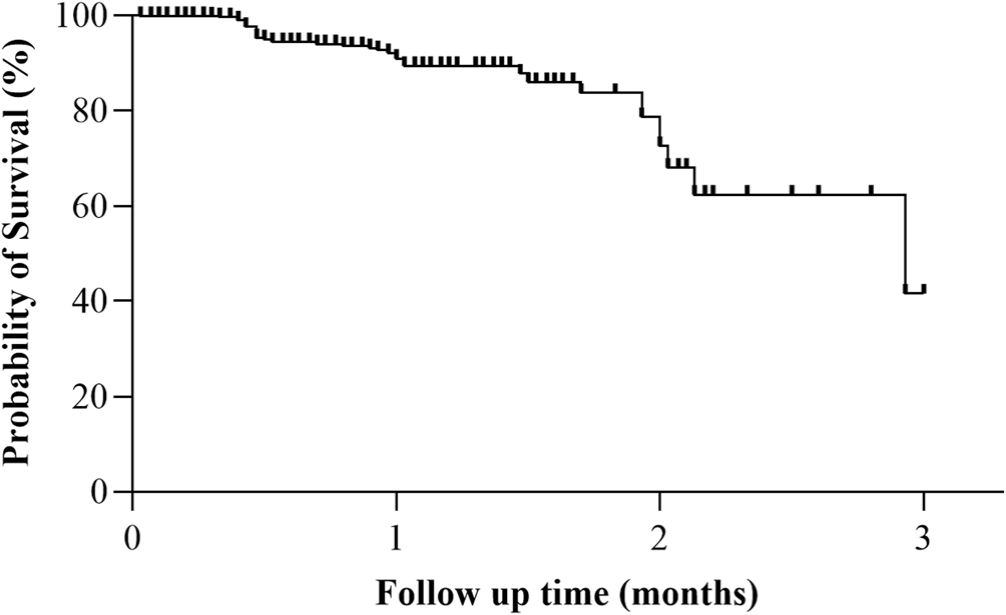
Kaplan–Meier plot for the time to Adverse Events.

A total of 56 AEs were analyzed during 578.97 person-months of follow-up. The overall incidence of occurrence of the first AEs was 96.7 per 1000 person-months (pm) [95% C.I 74.4–125.7]. Figure 4 shows the Kaplan–Meier plot for the time to events. The median time to the first AE with a 95% confidence interval was 87.9 (47.4 to 128.4) days (Fig. 4).

The highest incidence was observed among the elderly aged 60 years and above, individuals in WHO HIV Clinical Stage 2, individuals who were widowed/separated and individuals with a secondary level of education (Table 2).

**Table 2:** Incidence rate of occurrence of AEs across the population characteristics (N = 887). CI confidence interval.

Characteristics	Person-months (pm)	Event	AE incident rate per 1000 pm (95% CI)
Overall	578.97	56	96.7 (74.4–125.7)
Sex			
Male	205.5	20	97.3 (62.8–150.9)
Female	370.5	36	97.2 (70.1–134.7)
Age group (Years)			
12–19	18.4	2	108.9 (27.2–435.4)
20–24	62.1	9	144.9 (75.4–278.5)
25–59	467.5	41	87.7 (64.6–119.1)
≥ 60	26.4	4	**151.5 (56.9–403.7)**
Weight categories (In Kg)			
21–40	31.3	3	95.7 (30.9–296.9)
Above 40	504.4	50	99.1 (75.56–130.8)
Marital status			
Single	154.8	17	109.8 (68.3–176.7)
Cohabiting	26.8	4	149.4 (56.1–398.2)
Married	272.8	19	69.7 (44.4–109.2)
Divorced	55.8	4	71.6 (26.9–190.9)
Widow/Separated	56.9	9	**158.1 (82.2–303.8)**
Education level			
None	52.1	2	38.4 (9.6–153.5)
Primary	382.8	39	101.9 (74.4–139.4)
Secondary	121.3	13	**107.1 (62.2–184.5)**
College and above	20.2	2	38.4 (9.6–153.5)
Baseline WHO stage			
Stage 1	380.3	27	71.0 (48.7–103.5)
Stage 2	102.6	18	**175.4 (110.5–278.5)**
Stage 3	67.9	8	117.9 (59.0–235.7)
Stage 4	23.0	3	130.4 (42.1–404.4)
CD4 count at baseline (Cells/mm3)*			
< 50	9.5	0	–
50–< 200	78.4	9	114.7 (59.7–220.5)
200– < 500	194.9	22	112.9 (74.3–171,4)
≥ 500	10.6	0	–

### Risk factors for incidence rates of reported AEs

In the univariable analysis, the WHO HIV clinical stage was the significant predictor of reported AEs. After adjusting for potential confounders, WHO HIV clinical stage II, was the only independent risk factor for developing AEs within 90 days after initiating TLD (adjusted hazard ratio (aHR) = 2.73, 95% CI = 1.46–5.12) (Table 3).

**Table 3:** Predictors of incidence rates of reported AEs. cHR crude Hazard ratio, aHR adjusted Hazard ratio, CI confidence interval.

Characteristics	Univariate Cox proportional hazards model		Multivariate Cox proportional hazards model	
	cHR (95% CI)	*P*-value	aHR (95% CI)	*P*-value
Sex				
Male	1.06 (0.61–1.84)	0.83		
Female	1			
Age group (Years)				
12–19	0.97 (0.23–4.14)	0.58		
20–24	1.65 (0.80–3.39)			
25–59	1			
≥ 60	1.45 (0.51–4.13)			
Weight categories (Kg)				
20–40	1	0.92		
Above 40	0.95 (0.29–3.05)			
Marital status				
Single	1.56 (0.81–2.99)	0.31	1.51 (0.78–2.93)	0.20
Cohabiting	2.02 (0.68–5.95)		2.00 (0.67–6.02)	
Married	1		1	
Widowed/Separated	2.18 (0.98–4.84)		2.43 (1.08–5.45)	
Divorced	1.04 (0.35–3.07)		0.89 (0.30–2.68)	
Education level				
None	1	0.42	1	0.43
Primary	2.78 (0.67–11.59)		3.19 (0.72–14.11)	
Secondary	2.85 (0.64–12.68)		3.65 (0.76–17.59)	
College and above	2.95 (0.41–21.21)		3.97 (0.54–29.49)	
Baseline WHO HIV stage				
Stage 1	1	**0.042**	1	**0.017**
Stage 2	**2.47 (1.36–4.50)**		**2.73 (1.46–5.12)**	
Stage 3	1.52 (0.69–3.37)		1.46 (0.62–3.43)	
Stage 4	1.40 (0.41–4.76)		2.11 (0.60–7.43)	

Significant are in value [bold].

## Discussion

The major findings of this prospective study include a relatively low incidence of reported AEs suggesting that a DTG-based regimen is generally safe in the population; geriatrics and participants with WHO HIV clinical stage 2 were more likely to report AEs.

The proportion of participants who experienced at least one AE in this study was 6.3%. Our finding is relatively lower compared to that reported in other studies conducted among patients who also initiated DTG-based regimens in Brazil (10%), Kenya (18%)^25^ and Uganda (33%)^26^. Our study had a relatively larger sample size than these previous studies which might partly explain the observed differences, albeit other predisposing factors might also explain. The reported AEs were mild and included skin itching and rashes, nausea and vomiting, tiredness, diarrhoea, fever and stomach pain corroborating with several other studies^26^. Neuropsychiatric AEs were reported as one of the reasons for discontinuation of DTG-based regimens in previous studies^12,27^. However, in our present study, few individuals reported neuropsychiatric AEs which were mild and tolerable including headache, sleep disturbances and dizziness suggesting that AEs associated with treatment discontinuation may be less likely. The reason for the observed differences could be due to the design of the studies, or other predisposing factors across the different populations. Similar to our study, a previous study done in Switzerland reported a low proportion of neuropsychiatric AEs^28^.

DTG is also known to affect the level of cellular insulin interfering with lipid metabolism, resulting in obesity among patients^26^. In this study, few individuals had increased weight within three months of follow-up. Our study revealed that most reported AEs were classified as disorders affecting skin and subcutaneous tissues, gastrointestinal system, nervous system, metabolism and nutrition, respiratory and thoracic system according to MedDRA. Similar findings were reported from Brazil, and Zambia^29,30^. Several other studies are also in tandem with our findings^31–35^.

In our study, the overall incidence of occurrence of the first AE was 96.7 per 1000 person-months. This incidence rate is higher compared to 20–60 per 1000 person-months of ADRs among DTG users reported in previous studies^36–40^. However, our finding is lower than other studies conducted in South Africa and Congo^26,41,42^. The higher incidence observed in other studies might be due to longer follow-up times as compared to the present study. Furthermore, our study reports a lower median time for the first ADR (87.9 days) than a study in Brazil, which had a median time of occurrence of 210.6 days^25^.

The incidence rates of AEs were high in elderly participants aged 60 years and above and those who had a baseline WHO HIV clinical stage 2. The observed wide confidence intervals in the analysis may be explained by the few number of events observed. Nevertheless, this finding should be interpreted with caution as it may suggest little knowledge of the effect requiring further research. On further analysis, individuals on WHO HIV clinical stage 2 at baseline had significantly 2.7 higher AEs reported than those on stage 1. This finding is consistent with the findings from studies done in Brazil and Ethiopia^25,29,30^. Contrary to our finding, a study from Uganda reported that individuals who have initiated DTG-based regimens on lower stages 1, 2 and 3 were more likely to report AEs than those in stage 4. Individuals with advanced AIDS disease and elders may be more immune-compromised than those on stage 1, and younger, therefore, susceptible to developing AEs.

Our study had some limitations, which included a short period of follow-up which was 90 days. This might cause an underestimation of the actual effects of the DTG-based regimen. Nevertheless, our findings were similar to other studies with both longer and shorter follow-up times^36–40,43^, suggesting that the safety profiles of DTG-based regimens may not differ significantly with different times of follow-up. On the flip side, the strength of our study is the prospective design whereby AEs were reported as they occurred in real-world situations as well as involving many (n = 25) CTCs in 11 regions of the country.

We recommend continued monitoring of the safety of the DTG-based regimen in the population, particularly in elders and those with advanced HIV disease. Further studies should focus on the long-term effects of DTG on the liver, kidney and other systemic organs.

## Materials and methods

### Ethical statement

Ethical clearance was obtained from the National Institute for Medical Research (BA. 126/329/01A/85) and the study was performed according to relevant regulations and guidelines. Permission to conduct the study was obtained from the relevant region, districts and health facility authorities. Written informed consent and assent were obtained from all eligible participants. All data were encoded and treated confidentially, following data security principles and good clinical practices.

### Study design, population and setting

This was a prospective cohort study which involved PLHIV who were initiated on DTG based- regimen. The study included DTG naïve patients (newly ART initiated as well as clients who transitioned from another ART regimen to DTG). This study included individuals aged 12 years and above, newly diagnosed with HIV/AIDS and those initiated on or transitioned to Tenofovir, Lamivudine and Dolutegravir (TLD) during the study period. The study excluded seriously ill and hospitalized individuals. The study was conducted in 25 Care and Treatment Clinics (CTCs) from 11 regions of Tanzania's mainland. The regions were purposefully selected to represent administrative zones of the country, namely; the eastern zone (Morogoro, Dar es Salaam and Pwani), northern zone (Arusha and Tanga), lake zone (Shinyanga, Geita and Mwanza), central zone (Dodoma) and southern highlands zone (Iringa and Songwe). The CTCs were purposively selected by considering the number of PLHIV who were about to be initiated/transitioned to a DTG-based regimen. The CTCs with a relatively large number of PLHIV who were about to be initiated/transitioned to DTG-based regimen in each region were purposefully included. The list of CTCs and the corresponding number of individuals recruited is shown in Table 4.

**Table 4:** List of health facilities and the number of recruited individuals.

S/N	Health facilities	Individuals recruited	Region	Administrative zone
1	Kahama district hospital	50	Shinyanga	Lake zone
2	Shinyanga regional referral hospital	50	Shinyanga	
3	Geita regional referral hospital	50	Geita	
4	Katoro health center	50	Geita	
5	Sekou-Toure regional referral hospital	50	Mwanza	
6	Nyamagana hospital—district hospital	50	Mwanza	
7	Mwananyamala regional referral hospital	60	Dar es Salaam	Eastern Zone
8	Amana regional referral hospital	60	Dar es Salaam	
9	Mnazi Mmoja health center	50	Dar es Salaam	
10	Temeke regional referral hospital	60	Dar es Salaam	
11	Mbagala Rangi Tatu health center	50	Dar es Salaam	
12	Kilosa district hospital	60	Morogoro	
13	Bagamoyo district hospital	60	Pwani	
14	Mafinga district hospital	60	Iringa	Southern Highlands Zone
15	Iringa regional referral hospital	60	Iringa	
16	Ipamba designated hospital	60	Iringa	
17	Vwawa hospital	60	Songwe	
18	Tunduma town hospital	60	Songwe	
19	Dodoma regional referral hospital	100	Dodoma	Central Zone
20	Makole health center	100	Dodoma	
21	Ngamiani health center	60	Tanga	Northern Zone
22	Muheza DD hospital	60	Tanga	
23	Tanga regional referral hospital	60	Tanga	
24	St. Elizabeth council designated hospital	60	Arusha	
25	Mount Meru regional referral hospital	60	Arusha	
	**Total individuals recruited**	**1500**		

### Data collection

Data were collected electronically using structured questionnaires through the Open Data Kit (ODK®). Prior to data collection, healthcare workers from selected CTCs were recruited as research assistants and were trained on essential components of the study, including a thorough orientation of the tools (i.e., informed consent/assent and study questionnaires). The tool was pre-tested in the field, validated and finalized.

Baseline information collected were socio-demographic characteristics and clinical profiles of participants. The socio-demographic characteristics including sex, age, body weight, marital status and educational level were collected during recruitment; whereas clinical profile (WHO HIV clinical stage, presence of any comorbidities, CD4 cell count and treatment history) were abstracted from the client’s medical records. The adverse events experienced by study participants anytime within 90 days of TLD initiation were also collected at the CTCs during scheduled visits. In all participants (both initiated and transitioned to DTG-based regimen), the visits followed the routine standard of care, at day 14, day 30 and day 90 since drug initiation.

### Study outcomes

The primary endpoint was the time to occurrence of any AE after initiation of DTG-based regimen. At enrollment, a thorough assessment of any presenting symptoms was done for all individuals. The reported AE was only considered DTG-based regimen associated if it was not reported at enrollment before drug initiation. Other secondary study outcomes were the types and severity of AEs. AEs were graded according to the internationally regognized Common Terminology Criteria for Adverse Events (CTCAE) version 4. According to these criteria, AEs are graded as Mild (Grade 1: asymptomatic or mild symptoms; clinical or diagnostic observations only; intervention not indicated); Moderate (Grade 2: minimal symptoms; local or non-invasive intervention indicated; limiting age-appropriate instrumental activities of daily living) or Severe (Grade 3: medically significant but not immediately life-threatening; hospitalization or prolongation of hospitalization indicated; disabling; limiting self-care activities of daily living)^44^. The AEs were further classified according to the Medical Dictionary for Regulatory Activities (MedDRA) which is a clinically validated international medical terminology dictionary-thesaurus used by regulatory authorities for safety information.

### Statistics and analysis

Data analysis was done using STATA 15 (College Station, TX). Categorical variables were summarized using frequencies with proportions and median with interquartile range (IQR) were used to summarize continuous variables. Univariable and multivariable Cox regression were used to investigate risk factors for the occurrence of AEs. Variables of clinical significance or with *p*-value ≤ 0.2 were added to the multivariable model. *P*-values < 0.05 were considered statistically significant.

## Data Availability

All data generated or analyzed during this study are included in this manuscript.
